# Multi-Target Restoration of Dermal Elastic Fibers Through Elastin Upregulation, Elastase Suppression, and Scaffold Reinforcement

**DOI:** 10.3390/cimb48050431

**Published:** 2026-04-22

**Authors:** Sanghyun Ye, Seongsu Kang, Eui Taek Jeong, Seung-Hyun Jun, Nae-Gyu Kang

**Affiliations:** R&I Research Division, LG Household and Health Care R&D Center, Seoul 07795, Republic of Korea; shye123@lghnh.com (S.Y.); kss6279@gmail.com (S.K.); etjeong@lghnh.com (E.T.J.)

**Keywords:** elastic fiber, elastin homeostasis, skin photoaging, microfibrillar scaffold

## Abstract

Elastic fibers are key components of the skin extracellular matrix and are essential for maintaining skin integrity and elasticity. During skin aging, particularly photoaging, elastic fiber integrity is progressively compromised by increased elastase activity and the downregulation of elastin and scaffold-related gene expression. Therefore, effective strategies to preserve elastic fiber function should address not only elastin synthesis but also enzymatic degradation and scaffold integrity. In this study, we investigated a multitarget approach to restoring the elastic fiber network by modulating elastin production, elastase activity, and scaffold protein expression. We found that Copper Tripeptide-1 enhanced elastin expression and secretion, ethyl ferulate inhibited elastase activity, and cedrol promoted scaffold-related gene expression and microfibrillar protein restoration in dermal fibroblasts. To assess the biological relevance of this approach, the combined treatment was evaluated using UV-damaged human skin biopsy samples. This combination effectively mitigated UV-induced elastic fiber disruption and significantly improved fiber architecture, as confirmed by immunofluorescence staining and scanning electron microscopy. These findings indicate that coordinated modulation of elastin production, proteolytic protection, and scaffold reinforcement is essential for maintaining elastic fiber integrity and represents a promising approach for preserving skin elasticity during aging.

## 1. Introduction

Loss of skin elasticity is a defining feature of intrinsic aging and is particularly pronounced in photoaging [1,2,3]. Chronically sun-exposed skin undergoes progressive structural degeneration and abnormal accumulation of elastic fibers, primarily driven by impaired elastin production and increased protein degradation [4,5]. These changes ultimately result in reduced elasticity and increased dermal laxity [2,5]. Unlike collagen, elastin exhibits an exceptionally low turnover rate in the adult skin, indicating that once elastic fibers are damaged, their capacity for spontaneous repair is extremely limited [6,7,8]. Owing to this intrinsic biological constraint, effective anti-aging strategies must focus on preserving elastic fiber integrity rather than relying on endogenous regeneration.

Elastic fibers are structurally complex assemblies composed of cross-linked elastin integrated into a microfibrillar matrix [6]. Importantly, elastin does not function independently; functional elastic fibers require precise spatial organization within a fibrillin-rich scaffold [9]. Proper formation and stabilization of elastic fibers require not only tropoelastin synthesis and cross-linking but also the coordinated participation of multiple accessory and linker proteins, including fibrillin-1-rich microfibrils, fibronectin, fibulins, and latent transforming growth factor-β-binding proteins (LTBPs) [10,11,12]. Fibronectin provides an initial scaffold that guides fibrillin-1 deposition, and the disruption of this process compromises microfibrillar organization [11,13]. In addition, fibulin-5 and LTBP-4 function as molecular chaperones that anchor tropoelastin to microfibrils, enabling the formation of linear, functional elastic fibers within the dermal matrix [12,14]. Accordingly, increased elastin synthesis alone is insufficient to restore elastic fiber functionality when scaffold integrity is compromised [15,16,17]. Therefore, strategies that reinforce this microfibrillar scaffold are mechanistically relevant for stabilizing and restoring elastin architecture.

Photoaging is characterized by elevated proteolytic activity that targets elastic fiber components [18,19]. Among the various proteolytic enzymes, macrophage- and dermal fibroblast-derived elastases play a central role in elastin degradation and photoelastosis development [18,20]. UV-induced upregulation of elastase activity accelerates elastic fiber fragmentation, further limiting the persistence of newly synthesized elastin [21]. Accordingly, pharmacological and plant-derived elastase inhibitors have been proposed to alleviate wrinkle formation by protecting elastic fiber integrity [22,23]. Therefore, sustainable enhancement of skin elasticity requires not only stimulation of elastin biosynthesis but also effective suppression of elastin degradation.

Collectively, these observations indicate that elastic fiber integrity is governed by a tightly interconnected regulatory network encompassing elastin biosynthesis, proteolytic balance, and scaffold-mediated assembly [24]. Disruption of any single component destabilizes the entire elastic fiber system [24]. However, many existing interventions have focused on single-target approaches—primarily increasing elastin synthesis or inhibiting elastase activity—without addressing the coordinated regulation required for durable elastic fiber restoration [23,25,26]. Consequently, improvements in elastin levels do not consistently translate into recovery of organized elastic fiber architecture, particularly under chronic photoaging conditions.

Based on the multifactorial nature of elastic fiber degeneration, we hypothesized that a coordinated multitarget approach could more effectively restore elastic fiber structure. Specifically, the simultaneous promotion of elastin synthesis, inhibition of elastase activity, and reinforcement of scaffold protein expression may collectively preserve elastic fiber integrity. To test this hypothesis, we selected Copper Tripeptide-1, ethyl ferulate, and cedrol based on their previously reported biological activities related to extracellular matrix regulation and skin aging [27,28,29]. Structurally, Copper Tripeptide-1 is a small, naturally occurring peptide complex (glycyl-L-histidyl-L-lysine bound to copper ions), which enables high bioavailability, efficient cellular uptake, and stability compared to larger biomolecules [30]. Consistent with these characteristics, Copper Tripeptide-1 has been extensively studied for its role in promoting extracellular matrix remodeling and wound healing [31]. Ethyl ferulate, a derivative of ferulic acid, is known for its potent antioxidant activity and protective effects against UV-induced oxidative stress [28]. Cedrol, a naturally occurring sesquiterpene, has been reported to influence dermal fibroblast function and extracellular matrix-related gene expression [29].

Accordingly, we first examined the effects of Copper Tripeptide-1 on elastin gene expression and secretion in dermal fibroblasts. We then assessed the elastase-inhibitory activity of ethyl ferulate using both cell-free and cell-based models. In addition, we evaluated the role of cedrol as a scaffold-reinforcing agent by analyzing its effects on the expression of key linker and microfibrillar proteins involved in elastic fiber assembly.

Finally, to determine the biological relevance of this multitarget strategy, we conducted an ex vivo study using human skin biopsy samples to evaluate the combined effects of Copper Tripeptide-1, ethyl ferulate, and cedrol on elastin content and dermal architecture under UV-induced damage. By integrating molecular, biochemical, and ultrastructural analyses, this study aims to establish a network-oriented dermal strategy for maintaining elastic fiber integrity under photoaging conditions.

## 2. Materials and Methods

### 2.1. Cell Culture and Preparation

Human dermal fibroblasts (Hs68) were obtained from the American Type Culture Collection (Manassas, VA, USA) and maintained in Dulbecco’s Modified Eagle’s Medium (DMEM; Gibco, Grand Island, NY, USA) supplemented with 10% fetal bovine serum (FBS; Gibco) and 1% penicillin–streptomycin (Gibco). Cells were incubated at 37 °C in a humidified atmosphere containing 5% CO_2_.

Copper Tripeptide-1 was purchased from Active Peptide Company (Cambridge, MA, USA). Ethyl ferulate and cedrol were obtained from Hanacare (Suwon, Republic of Korea) and Livetech Co., Ltd. (Seoul, Republic of Korea), respectively. For efficacy evaluation, each compound was used at a final concentration of 10 ppm unless otherwise specified.

All cell-based experiments were performed using at least three independent biological replicates.

### 2.2. Enzyme-Linked Immunosorbent Assay (ELISA)

Hs68 fibroblasts were seeded in 24-well plates at a density of 2 × 10^4^ cells per well and allowed to attach for 24 h. To assess the elastin-inducing activity of Copper Tripeptide-1, cells were treated with the test compounds prepared in serum-free DMEM for 24 h. For evaluation of UV-protective effects, cells were first exposed to a single dose of UV-B irradiation (20 mJ/cm^2^) using a BIO-SUN irradiation system (Vilber Lourmat, Marne La Vallee, France) and then treated with the test compounds for an additional 24 h.

Following treatment, culture supernatants were collected, and secreted elastin levels were measured using a Human Elastin (ELN) ELISA kit (Feiyue Bio, Beijing, China) according to the manufacturer’s instructions. Elastin concentrations were normalized to total protein content, which was quantified using the Pierce BCA Protein Assay Kit (Thermo Fisher Scientific, Waltham, MA, USA).

### 2.3. Gene Expression Analysis via Quantitative Real-Time PCR (RT-qPCR)

Hs68 fibroblasts were seeded in 6-well plates at a density of 3 × 10^5^ cells per well and incubated for 24 h. The cells were then exposed to the indicated test compounds in serum-free culture medium for an additional 48 h. Total RNA was extracted using the AccuPrep^®^ Universal RNA Extraction Kit (Bioneer, Daejeon, Republic of Korea) according to the manufacturer’s protocol. RNA quantity and purity were evaluated by measuring absorbance at 230, 260, and 280 nm using a NanoDrop spectrophotometer (Thermo Fisher Scientific, Waltham, MA, USA). For complementary DNA (cDNA) synthesis, 1 μg of total RNA was reverse-transcribed using a commercial cDNA synthesis kit (PhileKorea, Seoul, Republic of Korea). Reverse transcription was performed in a Veriti 96-well Thermal Cycler (Applied Biosystems, Foster City, CA, USA) at 42 °C for 30 min, followed by enzyme inactivation at 72 °C for 10 min. RT-qPCR was carried out using Power SYBR™ Green PCR Master Mix (Applied Biosystems) on a StepOnePlus™ Real-Time PCR System (Applied Biosystems). Gene-specific primers used for amplification are listed in Table 1. GAPDH was used as a housekeeping gene for normalization.

### 2.4. Elastase Activity Assay

Elastase inhibitory activity of ethyl ferulate was evaluated using the EnzChek™ Elastase Assay Kit (Thermo Fisher Scientific) following the manufacturer’s protocol with minor modifications. To assess the direct elastase inhibitory activity of ethyl ferulate, diluted ethyl ferulate solution (50 μL) was preincubated with porcine pancreatic elastase (0.5 U/mL, 100 μL) for 15 min at room temperature. Subsequently, 50 μL of DQ™ elastin substrate (0.1 mg/mL) was added to the reaction mixture. After incubation for 30 min in the dark, fluorescence intensity was measured using a microplate reader at an excitation wavelength of 485 nm and an emission wavelength of 538 nm. Elastase activity was calculated relative to that of enzyme-only control samples.

To evaluate the elastase inhibitory effect of ethyl ferulate in skin fibroblasts, Hs68 cells were seeded in 24-well plates and allowed to attach overnight. Cells were then exposed to a single dose of UV-B irradiation (20 mJ/cm^2^) and subsequently treated with ethyl ferulate diluted in serum-free medium for 24 h. Following treatment, culture supernatants were collected to determine extracellular elastase activity.

To assess cellular elastase activity, cells were washed with phosphate-buffered saline (PBS) and lysed using RIPA buffer. Cell lysates were centrifuged to remove debris, and the clarified supernatants were subjected to elastase activity measurement using the same assay kit. Fluorescence intensity was recorded as described above.

### 2.5. Immunofluorescence Staining for Linker Protein (Fibronectin-1)

Hs68s were seeded in 24-well plates at a density of 2 × 10^4^ cells per well and cultured until overconfluence for 7 days. Cells were treated with the cedrol (10 ppm) and incubated for an additional 24 h. Following treatment, cells were fixed with 4% paraformaldehyde (PFA) for 10 min at room temperature and washed three times with PBS. Cell membranes were permeabilized with 0.1% Triton X-100 in PBS for 10 min at room temperature, followed by three washes with PBS. Nonspecific binding was blocked by incubating the cells with 5% FBS in PBS for 30 min at room temperature.

For immunofluorescence analysis of linker proteins, cells were incubated overnight at 4 °C with primary antibodies against Fibronectin-1 (Abcam, Cambridge, UK, ab2413; 1:250) prepared in a blocking solution. After washing with PBS, the cells were incubated with goat anti-rabbit IgG secondary antibody conjugated to Alexa Fluor 488 (Invitrogen, a11034; 1:1000) for 1 h at room temperature in the dark. Nuclei were counterstained with DAPI (5 mg/mL stock solution diluted 1:1000 in PBS) for 10–20 min at room temperature in the dark. Cells were washed again with PBS, and fluorescence images were acquired using a fluorescence microscope (EVOS™ FL Auto2 Imaging System, Thermo Fisher Scientific). Images were analyzed using ImageJ software version 1.54 (NIH).

### 2.6. Immunohistochemical Staining of Human Skin Biopsies

Human skin biopsy specimens were purchased from Biopredic International (Rennes, France). According to the supplier, all tissues were obtained from donors who had provided informed consent and were collected in accordance with applicable ethical regulations for research use. Biopredic International is authorized by the relevant French authorities to collect, process, store, and distribute human biological materials for scientific purposes (Authorization No. AC-2023-5431; last approval date: 19 July 2023). The biopsies were maintained in a long-term skin culture medium (Biopredic International) and exposed to repeated UV irradiation to mimic cumulative UV-induced photoaging. The irradiation protocol consisted of 50 mJ/cm^2^ UV-A and 100 mJ/cm^2^ UV-B administered every 2 d, for a total of four irradiation cycles. Following UV exposure, Copper Tripeptide-1, ethyl ferulate, and cedrol were topically applied using a cream-based vehicle formulation. The base cream was prepared using a mixture of aqueous and lipid-phase components, including humectants, emulsifiers, and stabilizers such as glycerin, dipropylene glycol, carbomer, xanthan gum, squalene, caprylic/capric triglyceride, and various fatty alcohols and surfactants. The formulation protocol was adapted from established procedures developed by LG H&H (Seoul, Republic of Korea). Test formulations containing the active compounds were compared with a corresponding vehicle control lacking these ingredients.

After treatment, skin tissues were fixed in 4% PFA, processed, and embedded in paraffin blocks. Paraffin-embedded sections (3 μm thickness) were prepared and dried at 60 °C for 30 min. The sections were deparaffinized in xylene (three changes, 15 min each) and rehydrated through a graded ethanol series (100%, 95%, and 70%; two washes each for 5 min), followed by rinsing in tap water.

Antigen retrieval was performed by heating the sections in citrate buffer for 10 min using a pressure cooker, after which the slides were cooled under running water. Tissue permeabilization was carried out with 0.5% Triton X-100 for 15 min, followed by three washes with PBS for 5 min each. Non-specific binding sites were blocked with 1% bovine serum albumin for 1 h at room temperature.

The sections were incubated overnight at 4 °C with a primary antibody against elastin (ab9519; mouse, 1:200). After washing with PBS, Alexa Fluor 488-conjugated secondary antibodies (anti-mouse or anti-rabbit, 1:200; Invitrogen) were applied for 1 h at room temperature. Nuclei were counterstained with DAPI using the VECTASHIELD mounting medium (Vector Laboratories, Burlingame, CA, USA). Fluorescent images were acquired using the EVOS™ FL Auto2 imaging system and quantified with ImageJ software.

### 2.7. Scanning Electron Microscopy (SEM)

Human skin biopsy samples were fixed overnight at 4 °C in 2.5% glutaraldehyde prepared in 1× PBS. Samples were then washed twice with PBS for 20 min each, followed by an additional wash with 0.1 M sodium cacodylate buffer for 20 min. Post-fixation was performed using 2% osmium tetroxide in 0.1 M sodium cacodylate buffer for 2 h at room temperature. After post-fixation, samples were washed twice with 0.1 M cacodylate buffer for 20 min each and rinsed once with distilled water. Dehydration was performed through a graded ethanol series (30%, 50%, 70%, 80%, 90%, and 100%), with samples incubated for 20 min at each concentration, followed by an additional incubation in 100% ethanol. Samples were further dehydrated using a 1:1 mixture of ethanol and hexamethyldisilazane (HMDS), followed by two incubations in 100% HMDS for 1 h each. The samples were then air-dried overnight at room temperature in a fume hood. Dried samples were mounted on SEM stubs using carbon adhesive tape and coated with platinum using an ion sputter coater (Hitachi E-1045, Hitachi Co., Ibaraki, Japan). Sputter coating was performed at 15 mA for 30 s on the front surface and 15 mA for 20 s on the sides.

SEM was performed using a Teneo VS SEM system (FEI, Lincoln, NE, USA) operated in secondary electron mode. Images were acquired at a resolution of 3072 × 2048 pixels with an accelerating voltage of 5 kV, a working distance of approximately 10.0 mm, an emission current of 40 pA, and a dwell time of 5 µs.

SEM imaging was performed in collaboration with an external core facility (Korea Institute of Science and Technology, Seoul, Republic of Korea). The operator was blinded to sample identity during image acquisition, and comparable regions were consistently selected across samples to minimize selection bias.

### 2.8. Statistical Analysis

All data are presented as mean ± standard error of the mean. Statistical analyses were performed using GraphPad Prism version 6.07 (GraphPad Software, La Jolla, CA, USA), with one-way analysis of variance followed by Student’s *t*-test. A *p*-value < 0.05 was considered statistically significant.

## 3. Results

### 3.1. Copper Tripeptide-1 Enhances Elastin Gene Expression and Protein Secretion in UV-Exposed Dermal Fibroblasts

**Figure 1:**
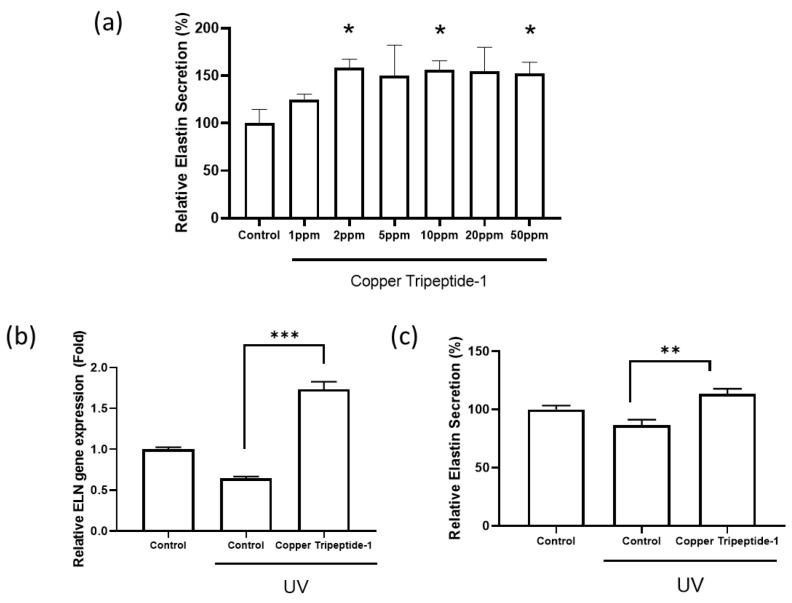
Copper Tripeptide-1 enhances elastin gene (*ELN*) expression and protein secretion. (**a**) Relative elastin secretion levels in Hs68 dermal fibroblasts treated with increasing concentrations of Copper Tripeptide-1 (*n* = 4). (**b**) Relative *ELN* mRNA expression levels in UV-B-exposed Hs68 fibroblasts with or without Copper Tripeptide-1 treatment (*n* = 4). (**c**) Relative elastin secretion in UV-B-irradiated Hs68 fibroblasts following Copper Tripeptide-1 treatment (*n* = 6). Error bars represent the standard error of the mean (SEM). * *p* < 0.05, ** *p* < 0.01, *** *p* < 0.001; one-way analysis of variance (ANOVA) and Student’s *t*-test.

To determine whether Copper Tripeptide-1 stimulates elastin synthesis, Hs68 dermal fibroblasts were treated with various concentrations (1–50 ppm), and elastin secretion was quantified using ELISA. As shown in Figure 1a, treatment increased elastin secretion in a dose-dependent manner up to 5 ppm, reaching approximately 150% of the control level.

To further examine its protective role against UV-induced damage, fibroblasts were exposed to UV irradiation and subsequently treated with Copper Tripeptide-1. RT-qPCR analysis revealed a significant increase in *ELN* gene expression compared to that with UV treatment alone (*p* < 0.001) (Figure 1b). Consistently, secreted elastin protein levels were also elevated following Copper Tripeptide-1 under UV-induced damage (*p* < 0.01) (Figure 1c).

### 3.2. Ethyl Ferulate Inhibits Elastase Activity in Cell-Free Systems and UV-Exposed Dermal Fibroblasts

**Figure 2:**
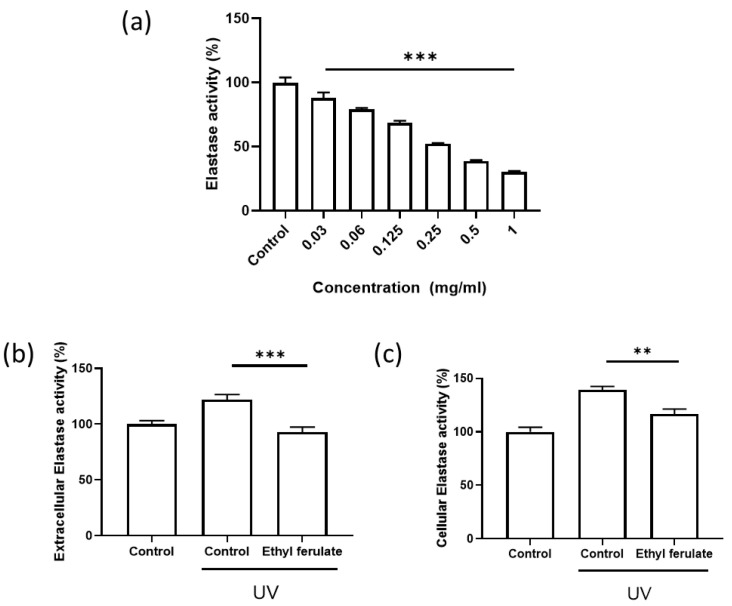
Ethyl ferulate inhibits elastase activity in cell-free and UV-exposed dermal fibroblast models. (**a**) Direct elastase-inhibitory activity of ethyl ferulate assessed using a cell-free enzymatic assay (*n* = 7). (**b**) Extracellular elastase activity measured in culture supernatants of UV-B-exposed Hs68 fibroblasts treated with ethyl ferulate (*n* = 8). (**c**) Cellular elastase activity assessed in lysates of UV-B-irradiated Hs68 fibroblasts following ethyl ferulate treatment (*n* = 8). Error bars represent the SEM. ** *p* < 0.01, *** *p* < 0.001; one-way ANOVA and Student’s *t*-test.

To evaluate the elastase-inhibitory effect of ethyl ferulate, elastase activity was assessed using a cell-free enzymatic assay and UV-exposed dermal fibroblast models. As shown in Figure 2a, ethyl ferulate suppressed elastase activity in a concentration-dependent manner in the cell-free system, indicating intrinsic elastase-inhibitory capacity.

Next, to determine whether ethyl ferulate inhibits elastase under photo-stressed conditions, Hs68 fibroblasts were exposed to UV-B irradiation and subsequently treated with ethyl ferulate. Extracellular elastase activity in culture supernatants was significantly increased following UV exposure, whereas ethyl ferulate treatment markedly reduced it (Figure 2b).

Consistently, intracellular elastase activity in fibroblast lysates was elevated following UV irradiation, and this increase was significantly attenuated upon ethyl ferulate treatment (Figure 2c). Collectively, these results demonstrate that ethyl ferulate effectively inhibits elastase activity, both in a cell-free system and within UV-damaged dermal fibroblasts, supporting its protective role against elastin degradation.

### 3.3. Cedrol Enhances Linker Protein Expression and Restores the Microfibrillar Scaffold in UV-Exposed Dermal Fibroblasts

**Figure 3:**
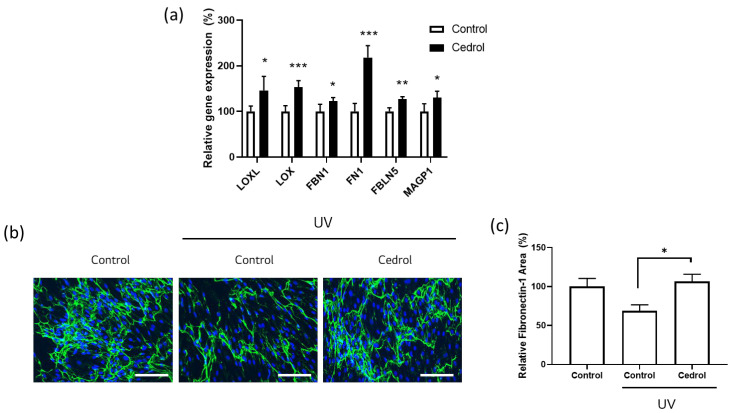
Cedrol enhances scaffold-related gene expression and restores microfibrillar proteins in UV-exposed dermal fibroblasts. (**a**) Relative mRNA expression levels of scaffold-related genes (*LOXL*, *LOX*, *FBN1*, *FN1*, *FBLN5*, and *MAGP1*) in dermal fibroblasts treated with cedrol (*n* = 4–6). (**b**) Representative immunofluorescence images of Fibronectin-1 in dermal fibroblasts under control, UV-exposed, and UV-exposed with cedrol treatment conditions. Green indicates fibrillin-1, and blue indicates DAPI. Scale bar = 150 μm. (**c**) Quantification of Fibronectin-1-positive area from immunofluorescence images (*n* = 5). Error bars represent the SEM. * *p* < 0.05, ** *p* < 0.01, *** *p* < 0.001; one-way ANOVA and Student’s *t*-test.

To investigate whether cedrol affects proteins involved in elastic fiber scaffolding, gene expression levels of key linker proteins were first analyzed via RT-qPCR. As shown in Figure 3a, cedrol treatment significantly upregulated the mRNA expression of *LOXL*, *LOX*, *FBN1*, *FN1*, *FBLN5*, and *MAGP1*, indicating broad activation of genes associated with elastic fiber assembly and stabilization [32].

Next, the effects of cedrol on microfibrillar structure under photo-stressed conditions were evaluated using immunofluorescence staining in UV-exposed dermal fibroblasts. UV irradiation markedly reduced Fibronectin-1 signals, reflecting disruption of the elastic fiber scaffold. In contrast, cedrol treatment restored the expression and organization of Fibronectin-1 (Figure 3b).

Quantitative image analysis further confirmed that the Fibronectin-1-positive area was significantly decreased following UV exposure but was substantially recovered upon cedrol treatment (Figure 3c). These findings demonstrate that cedrol upregulates microfibril-associated protein expression, suggesting a potential role in attenuating UV-induced changes in elastic fiber-related components.

### 3.4. The Triple Complex Restores Elastin Content and Preserves Fiber Architecture in UV-Damaged Human Skin Biopsies

To determine whether simultaneous modulation of elastin synthesis, degradation, and scaffold reinforcement results in restoration of elastic fibers, a human skin biopsy model was employed. Skin samples were exposed to repeated UV irradiation and subsequently treated with the Triple Complex (Copper Tripeptide-1, ethyl ferulate, and cedrol). Elastin distribution and structural integrity were evaluated via immunofluorescence staining and SEM.

**Figure 4:**
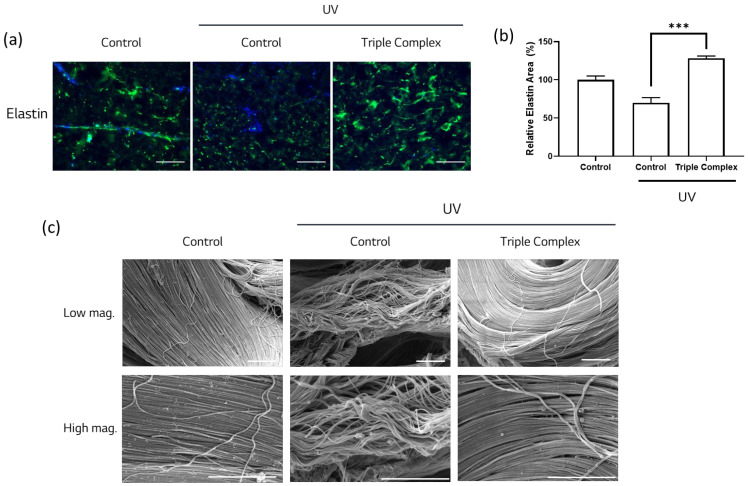
The Triple Complex (Copper Tripeptide-1, ethyl ferulate, and cedrol) restores elastin content and preserves fiber architecture in UV-damaged human skin biopsies. (**a**) Representative immunofluorescence images of elastin in human skin biopsies. Green indicates elastin, and blue indicates DAPI. Scale bar = 125 μm. (**b**) Quantification of elastin-positive area from immunofluorescence images (*n* = 5). (**c**) Representative scanning electron microscopy images of elastic fiber structures in human skin biopsies at low and high magnification. Scale bar = 5 μm. Error bars represent the SEM. *** *p* < 0.001; one-way ANOVA.

As shown in Figure 4a, UV exposure markedly reduced elastin fluorescence intensity and induced fragmented, dot-like elastin patterns, indicating disruption of elastic fiber organization. In contrast, treatment with the Triple Complex restored elastin signal intensity and promoted a more continuous and organized elastin distribution.

Quantitative image analysis further demonstrated that the elastin-positive area was significantly decreased following UV exposure, whereas treatment with the Triple Complex resulted in significant recovery of the elastin area compared with UV-treated controls (Figure 4b).

Structural changes in the fibers were further examined using SEM analysis. The UV-treated skin samples exhibited disrupted and loosely organized fiber structures, reflecting compromised dermal architecture. In contrast, skin biopsies treated with the Triple Complex displayed a dense and well-organized fiber network comparable to that observed in non-UV-exposed control samples (Figure 4c). These findings indicate that the Triple Complex effectively restores both elastin content and elastic fiber architecture in UV-damaged human skin.

## 4. Discussion

Degeneration of elastic fibers is a key feature of intrinsic aging and photoaging; however, effective strategies to restore their integrity remain limited [1,2,3]. Unlike collagen, elastin has a limited turnover rate and relies on a highly organized microfibrillar structure for functional assembly [6,7,8]. Consequently, disruption of elastin biosynthesis, increased proteolytic degradation, and collapse of scaffold architecture collectively contribute to the loss of skin elasticity [24]. In this study, we demonstrated that Copper Tripeptide-1 enhances elastin gene expression and secretion, ethyl ferulate suppresses elastase activity, and cedrol promotes the expression of scaffold-related proteins in dermal fibroblasts. Furthermore, we showed that the combined application of these compounds effectively protects elastic fiber architecture against UV-induced damage in human skin biopsy models.

Despite the strengths of this study, several limitations should be acknowledged. Although we demonstrated that each active compound modulated different aspects of elastic fiber homeostasis—namely elastin expression, elastase inhibition, and scaffold protein upregulation—the precise molecular mechanisms underlying these effects have not yet been investigated. The observed effects may be associated with complementary signaling mechanisms. Copper Tripeptide-1 and cedrol may act, at least in part, through MAPK signaling pathways that regulate extracellular matrix synthesis and cellular responses, while ethyl ferulate may reduce oxidative stress and suppress proteolytic activity via AMPK/Nrf2 signaling pathways [29,33,34]. Importantly, the concurrent regulation of these pathways may provide a coordinated control of elastic fiber homeostasis by simultaneously promoting elastin production and limiting its degradation, thereby supporting the maintenance of structurally and functionally competent elastic fibers under UV-induced stress. However, further studies are required to elucidate the precise molecular mechanisms underlying these interactions.

In this study, immunofluorescence staining was performed under overconfluent culture conditions to evaluate extracellular matrix (ECM)-associated linker proteins in a physiologically relevant environment. Under these conditions, cedrol treatment significantly enhanced the deposition of fibronectin-1, a key ECM glycoprotein that plays a critical role in fiber assembly and dermal matrix integrity [35]. These findings suggest that cedrol promotes ECM fiber formation and dermal matrix organization. Future studies investigating additional ECM-associated linker proteins under overconfluent conditions would further elucidate the mechanisms underlying elastic fiber assembly. Moreover, although overconfluent cultures can provide a physiologically relevant model of ECM-rich environments, more physiologically relevant models—such as three-dimensional (3D) skin equivalents or ECM-based matrices—will be valuable for validating the effects of cedrol on extracellular matrix remodeling and skin architecture [36].

Although structural restoration and preservation of elastic fibers were clearly demonstrated using immunofluorescence staining and SEM, functional assessments of skin elasticity were not conducted. Therefore, whether the observed architectural improvements translate into enhanced or maintained biomechanical properties of the skin warrants further investigation. In addition, while structural changes were evaluated using imaging-based approaches, more quantitative biochemical measurements, such as desmosine content analysis, would further strengthen the evaluation of elastin integrity. Furthermore, although ex vivo human skin biopsy models provide physiologically relevant insights, confirming their efficacy in vivo will be necessary to fully establish translational relevance.

Lastly, elastin expression following UV exposure is known to involve alterations in splice variants. Previous studies have reported that UV irradiation decreases normal elastin mRNA while increasing the expression of alternatively spliced variants containing exon 26A, which are associated with impaired elastic fiber assembly [37]. In the present study, elastin mRNA was assessed using primers targeting a region in the C-terminal portion of the ELN transcript, which does not distinguish between splice variants. Therefore, the observed changes reflect total elastin expression rather than isoform-specific regulation. Further studies using isoform-specific approaches will be required to determine whether the observed modulation corresponds to functionally competent elastin.

In summary, this study identified distinct functional compounds targeting key determinants of elastic fiber integrity and demonstrated that their combined application effectively protected elastic fiber architecture against UV-induced damage in human skin biopsy samples. These findings establish a proof of concept for a multi-target, network-oriented strategy to maintain elastic fiber structure under photoaging conditions. Beyond its potential application in cosmetic formulations, this approach contributes to a broader understanding of elastic fiber biology and may inform future skin research aimed at maintaining dermal resilience during aging.

## Figures and Tables

**Table 1:** List of primer sequences designed for target mRNA.

Target mRNA	Forward Primer	Reverse Primer
GAPDH	CATGTTCGTCATGGGGTGAACCA	AGTGATGGCATGGACTGTGGTCAT
ELN	GGAGTTGGTGGCTTAGGAGT	TTAACTCCTGCTCCAGTGGG
FBN1	GACATCAATCTGTGCGGGTC	CAACACACTGGTTCCACTGG
FN1	CCCCATTCCAGGACACTTCT	TGCCTCCACTATGACGTTGT
FBLN5	ATGTGTGGATGTGGACGAGT	GGATCCAGGAACATTCGCAC
MAGP1	AGTTCCAGTTCCAGTCCCAG	TGAGACACTGTTTGCAAGGC
LOX	ATTTCTTACCCAGCCGACCA	ATCCCTGTGTGTGTGCAGTA
LOXL1	CCAGGCTGCTATGACACCTA	GTTGCATCTCACCACGTTGT

## Data Availability

Data supporting the findings of this study are available from the corresponding author upon request.

## Abbreviations

The following abbreviations are used in this manuscript:

ANOVA	Analysis of variance
DMEM	Dulbecco’s Modified Eagle’s Medium
ELISA	Enzyme-linked immunosorbent assay
FBS	Fetal bovine serum
LTBPs	Latent transforming growth factor-β-binding proteins
PBS	Phosphate-buffered saline
RT-qPCR	Quantitative real-time polymerase chain reaction
SEM	Scanning electron microscopy
